# Towards a Self-Powered Amperometric Glucose Biosensor Based on a Single-Enzyme Biofuel Cell

**DOI:** 10.3390/bios14030138

**Published:** 2024-03-08

**Authors:** Asta Kausaite-Minkstimiene, Algimantas Kaminskas, Galina Gayda, Almira Ramanaviciene

**Affiliations:** 1 NanoTechnas—Center of Nanotechnology and Materials Science, Institute of Chemistry, Faculty of Chemistry and Geosciences, Vilnius University, Naugarduko St. 24, LT-03225 Vilnius, Lithuania; algimantas.kaminskas@chgf.stud.vu.lt (A.K.); almira.ramanaviciene@chf.vu.lt (A.R.); 2 Department of Analytical Biotechnology, Institute of Cell Biology National Academy of Sciences of Ukraine (ICB NASU), Dragomanov St. 14/16, 79005 Lviv, Ukraine; galina.gayda@nas.gov.ua

**Keywords:** glucose biosensor, single-enzyme biofuel cell, self-powered biosensor, glucose oxidase, Prussian blue, gold nanoparticles, poly(1,10-phenanthroline-5,6-dione)

## Abstract

This paper describes the study of an amperometric glucose biosensor based on an enzymatic biofuel cell consisting of a bioanode and a biocathode modified with the same enzyme—glucose oxidase (GOx). A graphite rod electrode (GRE) was electrochemically modified with a layer of Prussian blue (PB) nanoparticles embedded in a poly(pyrrole-2-carboxylic acid) (PPCA) shell, and an additional layer of PPCA and was used as the cathode. A GRE modified with a nanocomposite composed of poly(1,10-phenanthroline-5,6-dione) (PPD) and gold nanoparticles (AuNPs) entrapped in a PPCA shell was used as an anode. Both electrodes were modified with GOx by covalently bonding the enzyme to the carboxyl groups of PPCA. The developed biosensor exhibited a wide linear range of 0.15–124.00 mM with an R^2^ of 0.9998 and a sensitivity of 0.16 μA/mM. The limit of detection (LOD) and quantification (LOQ) were found to be 0.07 and 0.23 mM, respectively. The biosensor demonstrated exceptional selectivity to glucose and operational stability throughout 35 days, as well as good reproducibility, repeatability, and anti-interference ability towards common interfering substances. The studies on human serum demonstrate the ability of the newly designed biosensor to determine glucose in complex real samples at clinically relevant concentrations.

## 1. Introduction

Enzymatic biofuel cells (EBFCs) are a subtype of biofuel cells (BFCs) that use purified enzymes as a renewable biocatalyst to produce energy from various organic materials formed during metabolic processes [1]. EBFCs, like BFCs, operate in mild conditions, including low temperatures and physiological pH. Enzymes are highly selective for their substrates, so it is not necessary to separate the bioanodic part of the EBFC from the biocathodic part. This makes it possible to simplify the construction of the EBFC and reduce its size. Furthermore, it has been demonstrated that the electrical power produced can be increased by connecting several EBFCs in series. Thanks to the mentioned advantages and good biocompatibility, as well as the application of EBFCs in the fields of implantable energy supply devices and EBFC-based biosensors, they are attracting more and more attention from scientists all over the world [2]. Compared to conventional enzymatic electrochemical biosensors, EBFC-based biosensors have two main advantages: they have a simple design of only two electrodes and they do not require a battery or external power source [3] because by using the analyte as fuel, they can provide themselves with energy and simultaneously determine the concentration of the analyte. The two-electrode system and the absence of an external power source greatly simplify the required electronics and enable the development of miniature EBFC-based biosensors [4]. The sensing information of these biosensors can be transmitted in wireless mode with the help of a radio transmitter, so they can also be used as implanted remote EBFC-based self-powered biosensors (EBFC-SPBs) that allow for continuous monitoring of the change in the concentration of a certain analyte in the human body. EBFCs and EBFC-SPBs have already been implanted into living organisms such as cockroaches [5], clams [6], snails [7], rats [8], or rabbits [9]. It is likely that in the future, implanted EBFC-SPBs will be used to monitor blood glucose levels in diabetic patients, detect various substances that cause cancer or heart disease [10], and monitor human physiological changes [11]. The first EBFC-SPB was developed by Katz and their co-workers in 2001 [12]. It was able to detect glucose or lactate in a concentration range from 1 to 80 mM by measuring the variation in the output power depending on the concentration of the analyte. Since this work, the number of publications on EBFC-SPBs has been increasing every year and includes analytes such as ascorbic acid [13], lactate [14], glutathione [15], acetylcholine [16], ethanol [17], cholesterol [18], L-cysteine [19], mercury ions [20], or carcinoembryonic antigen [21]. However, EBFC-based glucose biosensors are of greatest interest. And this is not surprising, since more than 400 million people worldwide suffer from diabetes, a metabolic condition characterized by the body’s inability to control blood glucose levels. Monitoring and controlling glucose levels is essential to prevent serious complications from diabetes, such as heart attacks, strokes, kidney failure, blindness, or lower limb amputation. In addition to traditional methods of glucose measurement, EBFC-SPBs are a promising alternative for clinical patients who need to monitor their blood glucose levels daily. Glucose monitoring is also useful for adjusting medication, diet, and physical activity to achieve optimal blood glucose levels.

Most of the developed glucose EBFCs and EBFC-based biosensors use two enzymes: the anode is modified with a glucose-oxidizing enzyme, and the cathode is modified with an oxygen-reducing enzyme [22,23,24]. Bi-enzymatic biocathodes, in which a hydrogen peroxide-reducing enzyme is combined with a glucose-oxidizing enzyme, have also been described [25]. EBFCs and EBFC-based biosensors of this type have disadvantages such as the complexity of construction and increased costs due to the use of two different enzymes; each enzyme is usually characterized by certain operating conditions, and the difference in conditions may affect the performance. To overcome the second drawback and to improve performance, microfluidic devices using different and independent electrolytes suitable for the enzymes used have been developed [26]. However, the simplest way to avoid all the drawbacks is to use the same enzyme for anode and cathode modification. This principle was applied for the first time by Sekretova and their co-workers [27]. The anode of the single-enzyme EBFC-SPB (sEBFC-SPB) developed by these researchers was modified with the electron transfer mediator phenothiazine, and the cathode was modified with Prussian blue. Cholesterol oxidase was immobilized on the surface of both electrodes by using a sol–gel matrix. Under the action of this sEBFC-SPB, cholesterol oxidation catalyzed by phenothiazine-mediated cholesterol oxidase occurred at the bioanode surface. Meanwhile, hydrogen peroxide, a product of the enzymatic conversion of cholesterol on both bioelectrodes, was electrocatalytically reduced on the biocathode surface by Prussian blue. The sensitivity of sEBFC-SPB was much higher compared to either of the two individual electrodes, the dynamic range for cholesterol was from 0.15 to 4.1 mM, the liner range was from 0.15 to 0.52 mM, and the LOD was 1.4 μM. The obtained results showed the suitability of the developed sEBFC-SPB for the determination of cholesterol in human blood plasma. Later, Li and their co-workers developed an sEBFC-SPB for the detection of bisphenol A [28]. To develop it, laccase was encapsulated in a zeolitic imidazolate framework-8 and combined with bacterial cellulose and a carboxylated multi-walled carbon nanotube skeleton. In this way, highly flexible electrodes were prepared and were used as both biocathode and bioanode. The developed sEBFC-SPB exhibited an excellent linear range from 0.01 to 0.4 mM of bisphenol A, with an LOD of 1.95 × 10^−3^ mM.

Recently, our research group had reported a membrane-less single-enzyme BFC that was powered with glucose (sEGBFC) [29]. This sEGBFC was composed of two GREs. One of them was coated with a layer of PB nanoparticles embedded in a PPCA shell and an additional layer of PPCA synthetized by cyclic voltammetry (CV) (GRE/PB-PPCA/PPCA). The second electrode, also using CV, was modified with a nanocomposite composed of PPD and AuNPs entrapped in a PPCA shell (GRE/PPD/(AuNPs)PPCA). Both electrodes were then modified with GOx by covalently attaching the enzyme to PPCA via an amide bond, thereby preparing the GRE/PB-PPCA/PPCA–GOx biocathode and the GRE/PPD/(AuNPs)PPCA–GOx bioanode. The operation of the developed sEGBFC was based on the oxidation of glucose catalyzed by GOx on the bioanode surface and PB-electrocatalyzed the reduction of hydrogen peroxide, which was produced by an enzymatic reaction on both bioelectrodes, on a surface of the biocathode. It was observed that the open-circuit voltage and current density generated by the sEGBFC were directly proportional to the glucose concentration, and the sEGBFC exhibited good operational stability. Therefore, we came up with the idea to apply the developed sEGBFC as an amperometric glucose biosensor with a simplified design of only two electrodes and to investigate its main analytical characteristics as well as its ability to detect glucose in human serum samples. As a consequence, this work is devoted to the presentation of the results of the aforementioned studies and their detailed description.

## 2. Materials and Methods

### 2.1. Materials and Reagents

GOx (from *Aspergillus niger*, a lyophilized powder containing 360 U/mg protein), acetic acid (CH_3_COOH; 100%, Ph. Eur., extra pure), tannic acid (C_76_H_52_O_46_; ≥86%, p.a., ACS), potassium hexacyanoferrate (III) (K_3_[Fe(CN)_6_]; ≥99%, p.a., ACS), iron (III) chloride (FeCl_3_; ≥98.5%, anhydrous, extra pure), D(−)-fructose (C_6_H_12_O_6_; ≥99.5% purity), D(+)-galactose (C_6_H_12_O_6_; ≥98% purity), D(+)-mannose (C_6_H_12_O_6_; ≥99.5% purity), D(+)-xylose (C_6_H_12_O_6_; ≥99% purity), and N-(3-dimethylaminopropyl)-N′-ethylcarbodiimide hydrochloride (EDC-HCl; ≥99% purity) were obtained from Carl Roth GmbH (Karlsruhe, Germany). Graphite rods (150 mm length, 3.0 mm diameter, 99.999% purity), 1,10-phenanthroline-5,6-dione (C_12_H_6_N_2_O_2_; 97% purity), pyrrole-2-carboxylic acid (C_5_H_5_NO_2_; 99% purity), N-hydroxysuccinimide (C_4_H_5_NO_3_; 98% purity), L-ascorbic acid (C_6_H_8_O_6_; 99% purity), acetaminophen (CH_3_CONHC_6_H_4_OH; ≥99.0% purity), dopamine hydrochloride ((HO)_2_C_6_H_3_CH_2_CH_2_NH_2_ × HCl; ≥98% purity), sodium acetate (CH_3_COONa; ACS reagent, ≥99% purity), sodium dihydrogen phosphate monohydrate (NaH_2_PO_4_ × H_2_O; ACS reagent, ≥98% purity), disodium hydrogen phosphate (Na_2_HPO_4_; ACS reagent, ≥99.0% purity), hydrogen peroxide (H_2_O_2_; 30% (*w*/*w*) in water, with stabilizer), trisodium citrate dihydrate (HOC(COONa)(CH_2_COONa)_2_ × 2H_2_O; ACS reagent, ≥99.0% purity), uric acid (C_5_H_4_N_4_O_3_; ≥99.0% purity), and human serum (from human male AB plasma, USA origin, sterile-filtered) were purchased from Sigma-Aldrich Chemie GmbH (Taufkirchen, Germany). Hydrogen tetrachloroaurate (III) trihydrate (HAuCl_4_ × 3H_2_O; ACS reagent, 99.99% (metals basis), Au 49.0% min) was obtained from Alfa Aesar (Kandel, Germany), and ethanol absolute (CH_3_CH_2_OH; ≥99.8% purity) was from Honeywell (Charlotte, NC, USA). D-(+)-glucose monohydrate (C_6_H_12_O_6_ × H_2_O, ≥99.0% purity) and fuming hydrochloric acid (HCl; 37% purity) were acquired from Merck KGaA (Darmstadt, Germany). Sodium hydroxide (NaOH; pellets, Pharmpur, Ph Eur, BP, NF) was purchased from Scharlab S.L. (Sentmenat, Spain), and potassium chloride (KCl; for analysis, ACS, ISO, ≥99.5% purity) was from AppliChem GmbH (Darmstadt, Germany).

### 2.2. Preparation of Solutions

Solutions of 0.5 M pyrrole-2-carboxylic acid (PCA) and 9.52 mM 1,10-phenanthroline-5,6-dione (PD) were prepared in ethanol. Acetate-phosphate-buffered solution of 0.05 mM CH_3_COONa, 0.05 mM Na_2_HPO_4_ and 0.05 mM NaH_2_PO_4_ (APB), 0.1 M FeCl_3_, 0.1 M K_3_[Fe(CN)_6_], 1 M KCl, 1 M HCl, 0.1 M N-hydroxysuccinimide, 0.4 M EDC-HCl, and aqueous solutions of other materials were prepared using ultra-high-quality water. A 40.0 mg/mL GOx solution and standard solutions of 1.0 and 2.0 M glucose, 1.0 M fructose, 1.0 M galactose, 1.0 M mannose, 1.0 M xylose, 1.0 M uric acid, 1.0 M ascorbic acid, 1.0 M acetaminophen, and 1.0 M dopamine were prepared in APB supplemented with 0.1 M KCl (APB-KCl) with a pH of 6.0. The GOx solution was prepared immediately before use for the experiments. Solutions of glucose and other saccharides were prepared for at least 24 h before using them for experiments to allow for mutarotation to occur and to reach an equilibrium between α and β anomeric forms. The prepared solutions were stored at +4 °C, except for N-hydroxysuccinimide and EDC-HCl solutions, which were aliquoted in the required volumes into disposable test tubes and stored at −20 °C.

### 2.3. Preparation of Bioelectrodes and Current Measurements

The pre-treatment of GREs, synthesis of AuNPs, and preparation of biocathode and bioanode have been described in detail in our previously published articles [29,30,31]. For the biocathode preparation, a GRE was electrochemically modified with a layer of PB nanoparticles embedded in a PPCA shell (CV, 50 cycles from −0.4 to +1.0 V, scan rate of 0.1 V/s; synthesis solution: 100.0 mM HCl, 100.0 mM KCl, 1.0 mM FeCl_3_, 1.0 mM K_3_[Fe(CN)_6_] and 35.0 mM PCA) and then coated by the layer of PPCA (CV, 5 cycles from −0.4 to +1.0 V, scan rate of 0.1 V/s; synthesis solution: APB with 0.1 M KCl and 200.0 mM PCA). Accordingly, for the bioanode preparation, GRE was electrochemically modified with a nanocomposite composed of PPD and 13 nm AuNPs, entrapped in a PPCA shell (CV, 10 cycles from −0.4 to +1.0 V, scan rate of 0.1 V/s; 8.45 μg/cm^2^ PD on GRE; synthesis solution: 1.5 nM AuNPs, 100 mM PCA in APB-KCl solution). The carboxyl functional groups present on the surface of the biocathode and bioanode were activated with EDC/NHS (30 min) with the following 40 mg/mL GOx immobilization (30 min) on both electrodes. The biocathode was characterized by CV and it was able to generate s current density of 31.68 ± 2.70 μA/cm^2^ and an open-circuit potential (OCP) of 293.34 ± 15.70 mV in O_2_-saturated 10 mM glucose solution at pH 6.0, while the bioanode was characterized by CV and it was able to generate a current density of 8.63 ± 0.70 μA/cm^2^ and an OCP of 205.83 ± 5.10 mV.

The current generated by the developed sEGBFC-based biosensor was measured using a potentiostat/galvanostat Autolab PGSTAT30 (Eco Chemie, Utrecht, The Netherlands), which was controlled by a computer with specialized NOVA1.9 software. In a two-electrode electrochemical cell configuration, GRE/PPD/(AuNPs)PPCA–GOx was connected as the bioanode and GRE/PB-PPCA/PPCA–GOx as the biocathode. A potential difference of 0.3 V was maintained between the bioelectrodes during the measurements. All the measurements were carried out at an ambient temperature in oxygenated APB-KCl with a pH of 6.0. The content of the electrochemical cell was continuously stirred with a magnetic stirrer at 450 rpm. Bioelectrodes were stored in sealed containers over a drop of APB-KCl (pH 6.0) at +4 °C between experiments, and their surface was rinsed with ultra-high-quality water before each measurement.

### 2.4. Interpretation of Experimental Data

The current signal generated by the developed sEGBFC-based biosensor was expressed as the change in current (ΔI), which was calculated as the difference between the current observed when a certain amount of glucose standard solution was added to the electrochemical cell and the background current. The experimental results are presented as the average of three independent measurements, with standard deviations indicated by error bars.

The linear range of the current dependence on glucose concentration was determined from the calibration curves obtained by approximating the experimental data by the equation of a straight line (y = ax + b). In this way, the slope (a), intercept (b), and determination coefficient (R^2^) were also evaluated. The LOD was estimated as the concentration of glucose with a signal-to-noise ratio of 3, whereas the LOQ was estimated as the concentration with a signal-to-noise ratio of 10.

The response time of the developed sEGBFC-based biosensor was calculated as the time in seconds during which the current reached 90% of the steady-state value after a change in glucose concentration.

The repeatability and reproducibility of the generated current were assessed by calculating the relative standard deviation (RSD) of the experimental data obtained by analyzing five 10.0 mM glucose solutions using the same biosensor and five identically prepared biosensors, respectively. The RSD was expressed as a percentage and was obtained by multiplying the standard deviation of the data by 100 and dividing the resulting value by the average of the data.

The selectivity of the developed sEGBFC-based biosensor was assessed by the magnitude of the current response to other saccharides. For this, 10.0 mM of glucose was first added to the electrochemical cell and then, after the current had reached a constant value, other saccharides were added one after the other (10 mM of each) and the change in current was monitored. In the same way, the interference of some foreign substances was studied; however, in this case, 5.0 mM glucose, 0.5 mM uric acid, 0.2 mM ascorbic acid, 0.1 mM dopamine, and 0.2 mM acetaminophen were used.

The stability of the biosensor was evaluated by measuring the generated current response to glucose within 35 days. For this purpose, three biosensors were prepared under identical conditions and the currents generated were measured at the same time intervals. The magnitude of the current generated by each biosensor is expressed as a percentage of the current generated on the first day of the experiment. The results of the stability study are presented as the average of these three measurements, with the standard deviation shown as error bars.

The accuracy of the analysis of commercial human serum by the biosensor was evaluated in terms of recovery. A certain volume of serum with a known glucose concentration (determined with a glucometer FreeStyle Optium ART16648 Rev. B 05/10) was added to the electrochemical cell and after measuring the current generated by the biosensor, the glucose concentration was determined from a linear equation derived from a calibration curve constructed by analyzing glucose standard solutions. The serum was analyzed in triplicate, and the recovery in percentage was calculated by multiplying the detected concentration by 100 and dividing the resulting value by the actual concentration.

## 3. Results and Discussion

**Figure 1:**
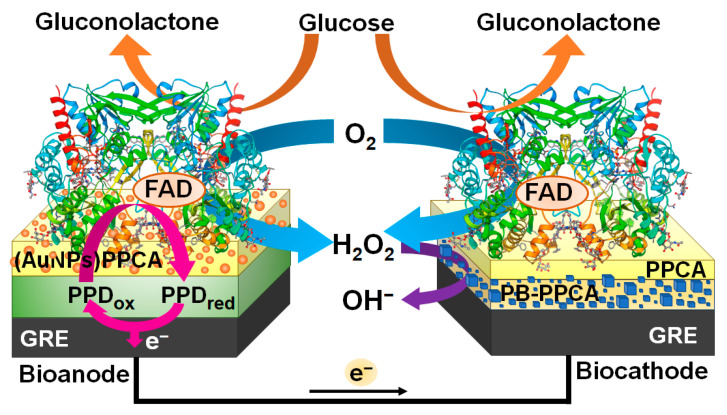
The structure and operation principle of the sEGBFC-based biosensor consisting of GRE/PB-PPCA/PPCA–GOx biocathode and GRE/PPD/(AuNPs)PPCA–GOx bioanode.

The amperometric glucose biosensor based on an enzymatic biofuel cell investigated and described in this work was constructed by coupling a bioanode and a biocathode modified with the same enzyme—GOx (Figure 1). A GRE electrochemically modified with a layer of PB nanoparticles embedded in a PPCA shell and an additional layer of PPCA was used as the cathode. Meanwhile, a GRE electrochemically modified with a nanocomposite composed of PPD and AuNPs entrapped in a PPCA shell was used as the anode. PPCA not only allowed for the covalent immobilization of GOx on the surface of both electrodes but also protected the surface from contamination due to its perm-selectivity properties. In the presence of glucose, PPD was able to transfer electrons generated during GOx-catalyzed glucose oxidation from the redox-active prosthetic group of the enzyme to the surface of the bioanode, and AuNPs were able to facilitate this electron transfer [31]. The presence of metallic species in the bioanode structure has been shown to increase its surface electronic conductivity and thus improve the electron transfer processes [32]. Meanwhile, PB nanoparticles on the surface of the biocathode exhibited electrocatalytic activity towards the reduction of hydrogen peroxide, which was generated during the enzymatic reaction occurring on both bioelectrodes [30]. The combination of these two bioelectrodes made it possible to develop the glucose biosensor based on the single-enzyme biofuel cell [29], of which the bioanode and biocathode were powered by the same substrate—glucose.

**Figure 2:**
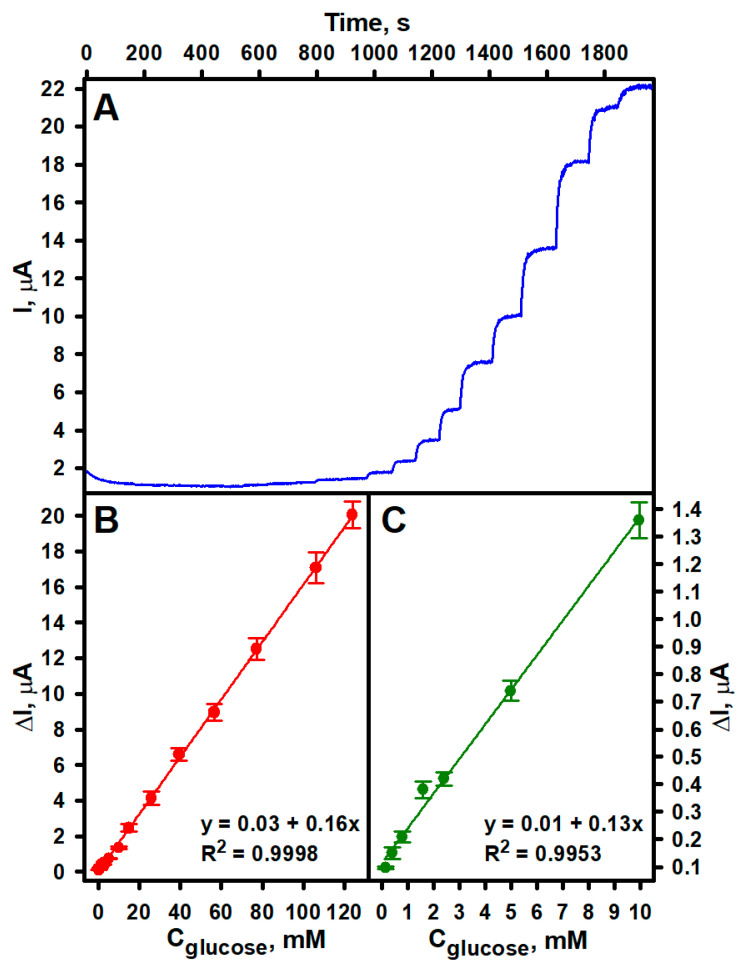
Dependence of the current generated by the developed sEGBFC-based biosensor on glucose concentration (**A**) and calibration curves at different intervals of glucose concentration (**B**,**C**). Measurement conditions: APB-KCl, pH 6.0; 0.3 V applied voltage.

In order to evaluate the biosensing capability of the developed sEGBFC-based biosensor, its current response was measured at various glucose concentrations. During the measurements, a constant potential difference of 0.3 V between the bioelectrodes was maintained, as the highest power density was determined at this potential [29]. The dependence of the current generated by the biosensor on the applied potential was also investigated, and the maximal current value was recorded at 0.3 V. The current increased with the increasing glucose concentrations (Figure 2A) in 5.0 mL of air-saturated APB-KCl solution, pH 6.0, which was stirred with a magnetic stirrer. As seen in Figure 2B, a linear relationship was determined over a wide range of glucose concentrations from 0.15 to 124.00 mM, with an R^2^ of 0.9998 (*N* = 3). The dependence of current at low glucose concentrations (from 0.15 to 9.95 mM) was presented in Figure 2C, and the calculated R^2^ was 0.9953 (*N* = 3). The response time in the presence of 4.99 mM glucose was 27 s. The fact that the current response is a function of glucose concentration indicates that the developed sEGBFC-based biosensor is suitable for glucose detection. The LOD and LOQ were found to be 0.07 and 0.23 mM, respectively, and the sensitivity was 0.16 μA/mM. According to the World Health Organization, the normal fasting blood glucose level is between 3.9 and 5.6 mM. When it reaches 5.6–6.9 mM, lifestyle changes and glucose monitoring are recommended. If fasting blood glucose is 7 mM or higher on two separate tests, diabetes is diagnosed. Meanwhile, if the fasting blood glucose level is less than 3.9 mM, hypoglycemia occurs. In this condition, it is also necessary to monitor the blood glucose level. Thus, the established linear range and LOQ are perfectly suited for the determination of glucose concentrations at hypoglycemic, normal, and hyperglycemic levels, as well as in other biofluids and industrial food samples. Comparing the developed sEGBFC-based biosensor with a conventional three-electrode amperometric biosensor with a GRE/PPD/(AuNPs)PPCA–GOx working electrode [31], a slightly lower LOD (0.07 mM in this work and 0.08 mM in a conventional three-electrode biosensor) was achieved. However, the linear range was slightly narrower (0.15–124.00 mM and 0.20–150.0 mM, respectively). Thus, the main advantages of the sEGBFC-based biosensor compared to the amperometric biosensor based on the GRE/PPD/(AuNPs)PPCA–GOx working electrode are the lower LOD, simpler construction, appropriate linear range for the practical application, and smaller size due to the absence of a third electrode. A comparison of the analytical parameters of the developed sEGBFC-based biosensor and some previously reported EBFC-based glucose biosensors is presented in Table 1. As can be seen from the examples presented, the analytical performance of the developed sEGBFC-based biosensor was similar, and the linear range was significantly wider. This significant extension of the linear range was achieved due to the use of PPCA in the biosensor design. This effect has already been observed and described for amperometric biosensors with conducting and non-conducting polymers, and the effect has been explained by hindered diffusion of the substrate, enzymatic reaction products, and the electron transfer mediator due to the polymer layer. In addition, the influence of the thickness of the polymer layer on the width of the linear range was observed. The thicker the layer, the stronger the diffusion limitations are and the wider the linear range of the biosensor is [33,34,35,36,37,38]. It is likely that these factors also affect the linear range of the developed sEGBFC-based biosensor. Diffusion limitations due to the PPCA layer on the surface of the bioelectrodes may also contribute to the longer response time of the biosensor. Although the obtained results are very encouraging, future research should focus on improving the design of the bioelectrodes and increasing the surface area-to-volume ratio of the bioelectrodes to increase sensitivity and reduce the LOD.

Reproducibility and repeatability are important parameters related to the performance of biosensors [47] and were therefore also investigated and evaluated. During the repeatability studies, the current response generated by one biosensor to 10.0 mM glucose was recorded five times one after the other. As can be seen from the experimental data presented in Table 2, only small differences in current were observed. The calculated RSD was 4.45%, indicating that the developed sEGBFC-based biosensor has good repeatability. The current response to 10.0 mM glucose of five identically prepared biosensors was measured during reproducibility studies. The developed sEGBFC-based biosensor also showed good reproducibility, giving almost the same current response in five independent experiments (Table 2), with an estimated RSD of 7.71%. The greater inaccuracy of different biosensors compared to the same biosensor could be caused by a small non-uniformity in the preparation of the bioelectrodes, during which a slightly different amount of PB, PPD, AuNPs, PPCA, or immobilized GOx was obtained on the surface.

**Figure 3:**
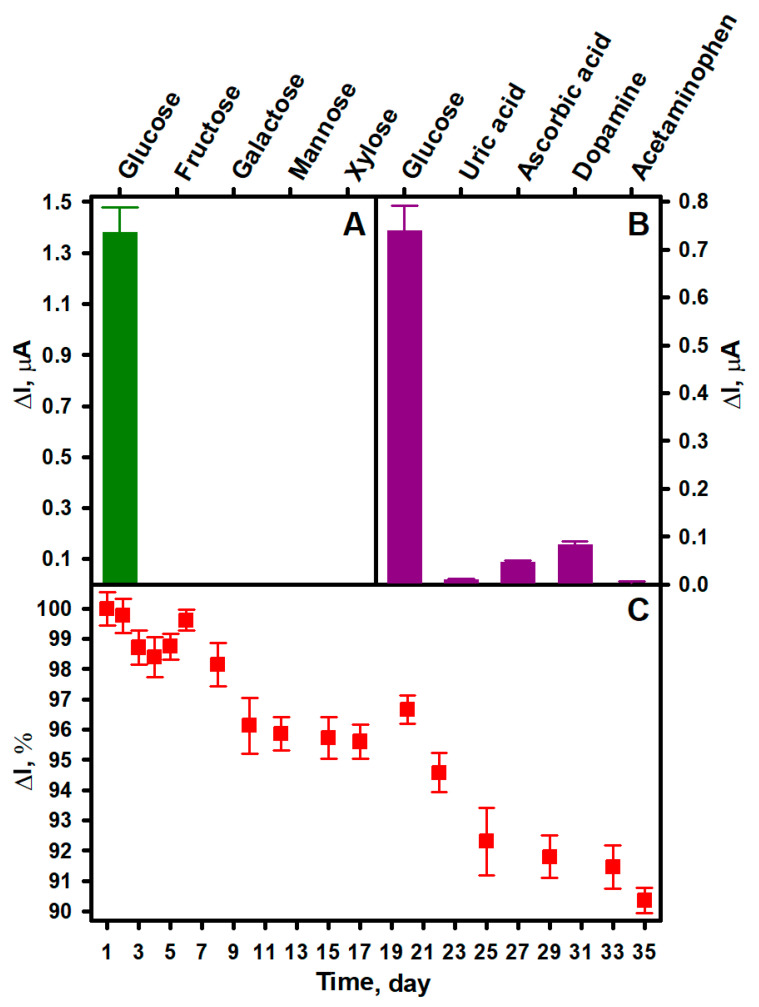
Comparison of the current response of the developed sEGBFC-based biosensor to glucose and other saccharides (**A**) and glucose and interfering substances (**B**). The current response of the developed sEGBFC-based biosensor vs. time (**C**). Measurement conditions: APB-KCl, pH 6.0; 10.0 mM glucose (**A**,**C**), fructose, galactose, mannose, and xylose (**A**); 5.0 mM glucose, 0.5 mM uric acid, 0.2 mM ascorbic acid, 0.1 mM dopamine, and 0.2 mM acetaminophen (**B**); 0.3 V applied voltage.

Although enzymes are highly selective for a certain analyte, their selectivity should be investigated when developing a new biosensor. It is important because the response of a biosensor is also influenced by its type (first, second, or third generation), the complexity of the sample, and the features of the biosensor construction, such as the type of electrode or surface modification [48]. Therefore, the selectivity of the developed sEGBFC-based biosensor for glucose, fructose, galactose, mannose, and xylose (at 10 mM of each) was also investigated. The experimental results presented in Figure 3A show that due to the high specificity and affinity of GOx for glucose, the biosensor is highly selective, as no current response was observed for the other tested saccharides. This presents the possibility of the practical application of developed biosensor.

Although the use of GOx as a biocatalyst in amperometric glucose biosensors and EBFCs is attractive due to the high specificity of this enzyme for glucose [42,49], their performance can be affected by the level of oxygen, as it is the natural electron acceptor of GOx. This is because variations in dissolved oxygen concentrations can cause changes in their response to glucose and affect the glucose detection range [50]. Electron transfer mediators are used to overcome this problem. If the electron transfer mediator is chosen appropriately, the electron transfer process becomes independent of the presence of natural electron acceptors or donors. In the case of the biosensor described in this work, the role of the electron transfer mediator played the PPD present in the bioanode structure. The application of PD in both GOx-based reagent-less glucose biosensors and EBFCs has been studied and described in several publications [31,51], and it has been found to be very suitable for electron transfer from GOx even in the presence of oxygen in the solution. Perhaps the most comprehensive study was carried out by Zor and their co-workers [52]. They developed an amperometric glucose biosensor whose working electrode was prepared by immobilizing GOx on a GRE electrode modified with carbon nanotubes and PD. The LOD value for this biosensor was found to be 8.0 mM in oxygen-saturated solution, and 10.7 mM in the free-of-dissolved-oxygen solution. Although the amperometric signal of the biosensor under anaerobic conditions was approximately 25% lower than under aerobic conditions, these data clearly demonstrate that PD is an efficient electron transfer mediator and the biosensor can be applied to glucose determination in real samples. On the other hand, GOx catalyzes the oxidation of glucose by molecular oxygen with the formation of gluconolactone and hydrogen peroxide, which is reduced on the surface of the biocathode of the sEGBFC-based biosensor under certain conditions. Thus, fluctuations in dissolved oxygen concentrations have some influence on both the biocathodic reaction and the magnitude of the current generated by the biosensor. A similar effect was described for the EBFC developed by Krikstolaityte and their co-workers [53]. The bioanode of this EBFC was modified with GOx, and the biocathode was modified with GOx and horseradish peroxidase. A significant decrease in the current generated by the biocathode was observed under anaerobic conditions because the lack of oxygen in the solution prevented the formation of hydrogen peroxide during the enzymatic reaction. The negative effect of the absence of oxygen on the registered signal magnitude was also observed for an amperometric glucose biosensor based on PB-functionalized TiO_2_ nanotube arrays developed and described by Gao and their co-workers [54]. Nevertheless, it was concluded that the used enzyme–TiO_2_ nanotube array integration strategy is promising for the development of biosensors and BFCs. Considering the results of the studies already performed, we have assumed that a similar influence would be observed for the sEGBFC-based biosensor developed in this work, so the influence of oxygen on the signal generated by it was not investigated.

Real samples without glucose may contain electroactive substances whose oxidation or reduction on the surface of the electrodes will affect the analytical signal of the biosensor and its applicability in practice. Some of the most common interfering substances are uric acid and ascorbic acid, as well as some pharmaceuticals [55] such as dopamine and acetaminophen. The normal concentration of uric acid in the serum of healthy adults is 0.15–0.45 mM (for women), while ascorbic acid ranges from 0.04 to 0.14 mM. The therapeutic concentration of acetaminophen in serum is 0.07–0.20 mM, and dopamine is present as less than 0.13 nM in ambulatory adults [56]. The influence of these substances on the current generated by the developed sEGBFC-based biosensor is shown in Figure 3B. The following concentrations were used in the study: 5.0 mM glucose, 0.5 mM uric acid, 0.2 mM ascorbic acid, 0.1 mM dopamine, and 0.2 mM acetaminophen. All the substances induced a current response, but these were much smaller compared to that induced by glucose. It was observed that dopamine had the greatest influence on the current generated by the biosensor and increased the current by 11.22%. Ascorbic acid, acetaminophen, and uric acid had lower influences at 6.35, 0.54, and 1.35%, respectively. This was probably due to the oxidation of these substances on the bioanode. However, considering the common concentrations of the tested substances mentioned above, the results of this study show that the developed sEGBFC-based biosensor has good anti-interference capability for these substances. A similar influence was observed for previously published glucose biosensors in which PPCA was applied. This influence was explained by the presence of negative charges resulting from the deprotonation of carboxyl groups, which can electrostatically repel negatively charged interfering substances and thus decrease their concentration at the electrode surface [31,56,57]. On the other hand, interfering substances often interfere at a potential of 0.6–0.7 V against the Ag/AgCl reference electrode [58], and a potential difference of 0.3 V between the bioelectrodes is probably insufficient for complete oxidation of the investigated substances, so their influence is small at normal concentrations.

Long-term stability is also a very important characteristic of biosensors, as the decay of the analytical signal over time would lead to false data of analysis [59]. Therefore, the stability of the developed sEGBFC-based biosensor was investigated by measuring its generated current response to 10.0 mM glucose daily or every few days over 35 days. Between measurements, the bioelectrodes were stored at +4 °C in sealed containers over a drop of APB-KCl (pH 6.0) to create a humid environment. The current generated by the biosensor gradually decreased, but as can be seen from Figure 3C, it retained 90.35% of the initial current after 35 days. The high stability of the developed sEGBFC-based biosensor can be primarily attributed to the covalent immobilization of GOx, which protects the enzyme from desorption from the surface of the bioelectrodes [4]. The hydrogen peroxide formed during GOx-catalyzed glucose oxidation affects the activity of the enzyme and therefore the stability of the biosensor [60]. However, the PB-electrocatalyzed reduction of hydrogen peroxide takes place on the biocathode surface of the sEGBFC-based biosensor, which reduces the influence of hydrogen peroxide on the decrease in enzyme activity. In addition, the high stability of the biosensor can also be related to the high stability of PB-PPCA/PPCA and PPD/(AuNPs)PPCA composites.

The potential practical application of the developed sEGBFC-based biosensor was tested by detecting glucose concentrations in commercial human serum. A serum with a known glucose concentration of 5.55 mM was used for analysis. During the analysis, the serum was injected into an electrochemical cell filled with 5 mL of APB-KCl (pH 6.0) in such a volume that the glucose concentration in the cell was 0.40 or 0.80 mM. Measurements were repeated three times with each glucose concentration. As can be seen from Table 3, the average recoveries of glucose with concentrations of 0.40 and 0.80 mM are 103.33 and 99.58%, respectively. And the RSDs of both samples are less than 6%. The determined recoveries and RSDs indicate that the developed sEGBFC-based biosensor can be used for the determination of glucose concentration in complex samples.

## 4. Conclusions

This paper describes a novel amperometric glucose biosensor based on an enzymatic biofuel cell consisting of a bioanode and a biocathode modified with the same enzyme. The operation of this biosensor is based on GOx-catalyzed oxidation of glucose on the bioanode and PB-electrocatalyzed reduction of hydrogen peroxide, which is formed on both bioelectrodes by enzymatic reaction, on the biocathode. The developed biosensor showed a wide linear range from 0.15 to 124.00 mM glucose with a sensitivity of 0.16 μA/mM, and an LOD and LOQ of 0.07 and 0.23 mM, respectively. The biosensor is very selective for glucose and had good reproducibility and repeatability as well as anti-interference ability towards common interfering substances. It has excellent operational stability and retained 90.35% of the initial current after 35 days. The studies on human serum demonstrate the ability of the developed biosensor to determine glucose in complex samples at clinically relevant concentrations. In addition, the simple design of only two electrodes allows for reducing the size of the biosensor and greatly simplifying the required electronics. Therefore, it is highly likely that the developed sEGBFC-based biosensor could be suitable as a self-powered biosensor. The design strategy of the developed biosensor could also be of great interest in the progress of biosensors for the detection of other analytes as well as in miniaturized power supplies for implantable medical devices. The current research is directed towards the application of the developed sEGBFC-based biosensor as a self-powered biosensor and the investigation of its ability to operate under physiological conditions.

## Figures and Tables

**Table 1:** Comparison of the analytical parameters of the developed sEGBFC-based biosensor and previously reported EBFC-based glucose biosensors.

Biocathode	Bioanode	Linear Range, mM	LOD, mM	LQD, mM	Stability, % from the Initial	Ref.
GRE/PB-PPCA/PPCA–GOx	GRE/PPD/(AuNPs) PPCA–GOx	0.15−124.0	0.07	0.23	90.35% after 35 days	this work
PGE/SWCNT/PBSE/BOx	PGE/SWCNT/PBSE/PQQ-GDH	up to 1.0	0.084	0.25	94% after 12 days	[39]
Cu	ITO/PB/GOx	0.10−1.20	14.34 × 10^−3^	−	>61% after 25 days	[40]
BP/PBA/CoPc/GOx	BP/PBA/MnO_2_	0.5−8.0	4.69 × 10^−3^	−	>82% after 31 days	[41]
ITO/MWCNTs/GOx	ITO/MWCNTs/Lac	up to 4.0	−	−	−	[42]
SPE/PBSE/BOx	SPE/Os/PQQ-GDH	0.1−0.6	−	−	7 days	[24]
BP/PBSE/BOx	BP/PBSE/PQQ-GDH	2.78–11.11	2.31	−	−	[22]
GCE/MWCNTs/ PBSE/HRP/GOx	GCE/rGO/poly(TBO)/NAD-GDH	0.1–0.7	−	−	−	[25]
BC/c-MWCNTs/AuNPs/Lac	BC/c-MWCNTs/AuNPs/GOx	0–50	2.87 × 10^−3^	−	60% after 30 days	[43]
Au/MWCNTs/Pt_plate_	Au/MWCNTs/PdNPs-PABA-GOx/CS	0.002–4.5	0.1 × 10^−3^	−	78.9% after 30 days	[44]
SPE/NiC/Nf	SPE/GOx/CS	1–5	−	−	−	[45]
Au/hPG/BOx	Au/hPG/OsPVI/PEGDGE/GOx	0.05−1.0	0.05	−	90% after 20 days	[46]

**Abbreviations:** PGE—pencil graphite electrode; PBSE—1-pyrenebutyric acid N-hydroxysuccinimide ester; SWCNT—single-walled carbon nanotubes; PQQ-GDH—pyrroloquinoline quinone-dependent glucose dehydrogenase; BOx—bilirubin oxidase; ITO—indium tin oxide glass; BP—buckypaper; PBA—1-pyrenebutyric acid; CoPc—cobalt phthalocyanine; MWCNTs—multi-walled carbon nanotubes; Lac—laccase; SPE—screen-printed electrode; Os—osmium-complex modified polymer; GCE—glassy carbon electrode; rGO—reduced graphene oxide; poly(TBO)—poly(toluidine-blue O); NAD-GDH—nicotinamide adenine dinucleotide-dependent glucose dehydrogenase; HRP—horseradish peroxidase; BC—bacterial cellulose; c-MWCNTs—carboxylic multi-walled carbon nanotubes; PdNPs—palladium nanoparticles; PABA—poly(3-anilineboronic acid); Pd_plate_—electroplated palladium; CS—chitosan; SPE—screen printed electrode; NiC—nickel with activated carbon catalyst; Nf—Nafion; hPG—highly porous gold; OsPVI—Os(2,2′-bipyridine)_2_ (polyvinylimidazole)_10_Cl]Cl; PEGDGE—polyethylene glycol diglycidyl ether.

**Table 2:** Data obtained during the study of repeatability and reproducibility.

Study	ΔI, µA	Average of ΔI, µA	STDEV	RSD, % (*N* = 5)
Reproducibility	1.40	1.25	0.10	7.71
	1.21			
	1.27			
	1.20			
	1.15			
Repeatability	1.40	1.33	0.06	4.45
	1.39			
	1.27			
	1.29			
	1.31			

**Table 3:** Determination of glucose concentration in human serum.

Added Glucose, mM	Detected Glucose, mM	Recovery, %	Average, %	RSD, % (*N* = 3)
0.40	0.42	105.00	103.33	5.04
0.40	0.43	107.50		
0.40	0.39	97.50		
0.80	0.79	98.75	99.58	3.83
0.80	0.77	96.25		
0.80	0.83	103.75		

## Data Availability

The data presented in this study are available on request from the first author.

## References

[1] Xiao X. (2022). The direct use of enzymatic biofuel cells as functional bioelectronics. eScience.

[2] Wang L., Wu X., Su B.S.Q., Song R., Zhang J.-R., Zhu J.-J. (2021). Enzymatic biofuel cell: Opportunities and intrinsic challenges in futuristic applications. Adv. Energy Sustain. Res..

[3] Hao S., Sun X., Zhang H., Zhai J., Dong S. (2020). Recent development of biofuel cell based self-powered biosensors. J. Mater. Chem. B.

[4] Gonzalez-Solino C., Bernalte E., Royo C.B., Bennett R., Leech D., Di Lorenzo M. (2021). Self-powered detection of glucose by enzymatic glucose/oxygen fuel cells on printed circuit boards. ACS Appl. Mater. Interfaces.

[5] Rasmussen M., Ritzmann R.E., Lee I., Pollack A.J., Scherson D. (2012). An implantable biofuel cell for a live insect. J. Am. Chem. Soc..

[6] Szczupak A., Halamek J., Halamkova L., Bocharova V., Alfonta L., Katz E. (2012). Living battery–biofuel cells operating in vivo in clams. Energy Environ. Sci..

[7] Halamkova L., Halamek J., Bocharova V., Szczupak A., Alfonta L., Katz E. (2012). Implanted biofuel cell operating in a living snail. J. Am. Chem. Soc..

[8] Cinquin P., Gondran C., Giroud F., Mazabrard S., Pellissier A., Boucher F., Alcaraz J.-P., Gorgy K., Lenouvel F., Mathe S. (2010). A glucose biofuel cell implanted in rats. PLoS ONE.

[9] Ichi-Ribault S.E., Alcaraz J.-P., Boucher F., Boutaud B., Dalmolin R., Boutonnat J., Cinquin P., Zebda A., Martin D.K. (2018). Remote wireless control of an enzymatic biofuel cell implanted in a rabbit for 2 months. Electrochim. Acta.

[10] Chen Y., Ji W., Yan K., Gao J., Zhang J. (2019). Fuel cell-based self-powered electrochemical sensors for biochemical detection. Nano Energy.

[11] Xue Z., Wu L., Yuan J., Xu G., Wu Y. (2023). Self-powered biosensors for monitoring human physiological changes. Biosensors.

[12] Katz E., Buckmann A.F., Willner I.J. (2001). Self-powered enzyme-based biosensors. Am. Chem. Soc..

[13] Zloczewska A., Celebanska A., Szot K., Tomaszewska D., Opallo M., Jonsson-Niedziolka M. (2014). Self-powered biosensor for ascorbic acid with a Prussian blue electrochromic display. Biosens. Bioelectron..

[14] Baingane A., Slaughter G. (2017). Self-powered electrochemical lactate biosensing. Energies.

[15] Roy B.G., Rutherford J.L., Weaver A.E., Beaver K., Rasmussen M. (2020). A self-powered biosensor for the detection of glutathione. Biosensors.

[16] Moreira F.T.C., Sale M.G.F., Lorenzo M.D. (2017). Towards timely Alzheimer diagnosis: A self-powered amperometric biosensor for the neurotransmitter acetylcholine. Biosens. Bioelectron..

[17] Ruff A., Pinyou P., Nolten M., Conzuelo F., Schuhmann W. (2017). A self-powered ethanol biosensor. Chem. Electro. Chem..

[18] Quah T., Abdellaoui S., Milton R.D., Hickey D.P., Minteer S.D. (2017). Cholesterol as a promising alternative energy source: Bioelectrocatalytic oxidation using NAD-dependent cholesterol dehydrogenase in human serum. J. Electrochem. Soc..

[19] Hou C., Fan S., Lang Q., Liu A. (2015). Biofuel cell based self-powered sensing platform for L-cysteine detection. Anal. Chem..

[20] Wen D., Deng L., Guo S., Dong S. (2011). Self-powered sensor for trace Hg^2+^ detection. Anal. Chem..

[21] Wang Y., Ge L., Wang P., Yan M., Yu J., Ge S. (2014). A three-dimensional origami-based immuno-biofuel cell for self-powered, low-cost, and sensitive point-of-care testing. Chem. Commun..

[22] Slaughter G., Kulkarni T. (2019). Detection of human plasma glucose using a self-powered glucose biosensor. Energies.

[23] Becker J.M., Lielpetere A., Szczesny J., Bichon S., Gounel S., Mano N., Schuhmann W. (2023). Wiring of bilirubin oxidases with redox polymers on gas diffusion electrodes for increased stability of self-powered biofuel cells-based glucose sensing. Bioelectrochemistry.

[24] Pinyou P., Conzuelo F., Sliozberg K., Vivekananthan J., Contin A., Poller S., Plumere N., Schuhmann W. (2015). Coupling of an enzymatic biofuel cell to an electrochemical cell for self-powered glucose sensing with optical readout. Bioelectrochemistry.

[25] Chansaenpak K., Kamkaew A., Lisnund S., Prachai P., Ratwirunkit P., Jingpho T., Blay V., Pinyou P. (2021). Development of a sensitive self-powered glucose biosensor based on an enzymatic biofuel cell. Biosensors.

[26] Escalona-Villalpando R.A., Sandoval-Garcia A., Espinosa L.J.R., Miranda-Silva M.G., Arriaga L.G., Minteer S.D., Ledesma-Garcia J. (2021). A self-powered glucose biosensor device based on microfluidics using human blood. J. Power Sources.

[27] Sekretaryova A.N., Beni V., Eriksson M., Karyakin A.A., Turner A.P.F., Vagin M.Y. (2014). Cholesterol self-powered biosensor. Anal. Chem..

[28] Li X., Li D., Zhang Y., Lv P., Feng Q., Wei Q. (2020). Encapsulation of enzyme by metal-organic framework for single-enzymatic biofuel cell-based self-powered biosensor. Nano Energy.

[29] Kausaite-Minkstimiene A., Kaminskas A., Ramanaviciene A. (2022). Development of a membraneless single-enzyme biofuel cell powered by glucose. Biosens. Bioelectron..

[30] Kausaite-Minkstimiene A., Kaminskas A., Popov A., Ramanavicius A., Ramanaviciene A. (2021). Development of a new biocathode for a single enzyme biofuel cell fuelled by glucose. Sci. Rep..

[31] Kausaite-Minkstimiene A., Glumbokaite L., Ramanaviciene A., Ramanavicius A. (2020). Reagent-less amperometric glucose biosensor based on nanobiocomposite consisting of poly(1,10-phenanthroline-5,6-dione), poly(pyrrole-2-carboxylic acid), gold nanoparticles and glucose oxidase. Microchem. J..

[32] Neto S.A., Milton R.D., Crepaldi L.B., Hickey D.P., de Andrade A.R., Minteer S.D. (2015). Co-immobilization of gold nanoparticles with glucose oxidase to improve bioelectrocatalytic glucose oxidation. J. Power Sources.

[33] Yang Y.-L., Tseng T.-F., Yeh J.-M., Chen C.-A., Lou S.-L. (2008). Performance characteristic studies of glucose biosensors modified by (3-mercaptopropyl)trimethoxysilane sol–gel and non-conducting polyaniline. Sens. Actuators B Chem..

[34] Kausaite-Minkstimiene A., Mazeiko V., Ramanaviciene A., Ramanavicius A. (2010). Enzymatically synthesized polyaniline layer for extension of linear detection region of amperometric glucose biosensor. Biosens. Bioelectron..

[35] Sharma A., Kumar A. (2016). Study of structural and electro-catalytic behaviour of amperometric biosensor based on chitosan/polypyrrole nanotubes-gold nanoparticles nanocomposites. Synth. Met..

[36] Mazeiko V., Kausaite-Minkstimiene A., Ramanaviciene A., Balevicius Z., Ramanavicius A. (2013). Gold nanoparticle and conducting polymer–polyaniline–based nanocomposites for glucose biosensor design. Sens. Actuators B Chem..

[37] Olea D., Viratelle O., Faure C. (2008). Polypyrrole-glucose oxidase biosensor: Effect of enzyme encapsulation in multilamellar vesicles on analytical properties. Biosens. Bioelectron..

[38] Ramanavicius S., Ramanavicius A. (2021). Conducting polymers in the design of biosensors and biofuel cells. Polymers.

[39] Torrinha A., Tavares M., Delerue-Matos C., Morais S. (2021). A self-powered biosensor for glucose detection using modified pencil graphite electrodes as transducers. Chem. Eng. J..

[40] Zhang Y., Hao S., Sun X., Zhang H., Ma Q., Zhai J., Dong S. (2022). A self-powered glucose biosensor based on mediator-free hybrid Cu/glucose biofuel cell for flow sensing of glucose. Electroanalysis.

[41] Hao S., Zhang H., Sun X., Zhai J., Dong S. (2021). A mediator-free self-powered glucose biosensor based on a hybrid glucose/MnO_2_ enzymatic biofuel cell. Nano Res..

[42] Khan H., Choi J.H., Ullah A., Kim Y.H., Kim G.M. (2020). Continuous determination of glucose using a membraneless, microfluidic enzymatic biofuel cell. Micromachines.

[43] Lv P., Zhou H., Mensah A., Feng Q., Wang D., Hu X., Cai Y., Lucia L.A., Li D., Wei Q. (2018). A highly flexible self-powered biosensor for glucose detection by epitaxial deposition of gold nanoparticles on conductive bacterial cellulose. Chem. Eng. J..

[44] Sun L., Ma Y., Zhang P., Chao L., Huang T., Xie Q., Chen C., Yao S. (2015). An amperometric enzyme electrode and its biofuel cell based on a glucose oxidase-poly(3-anilineboronic acid)-Pd nanoparticles bionanocomposite for glucose biosensing. Talanta.

[45] Fischer C., Fraiwan A., Choi S. (2016). A 3D paper-based enzymatic fuel cell for self-powered, low-cost glucose monitoring. Biosens. Bioelectron..

[46] Baingane A., Narayanan J.S., Slaughter G. (2018). Sensitive electrochemical detection of glucose via a hybrid self-powered biosensing system. Sens. Bio-Sens. Res..

[47] Giorgi G., Tonello S. (2022). Wearable biosensor standardization: How to make them smarter. Standards.

[48] Bucur B., Purcarea C., Andreescu S., Vasilescu A. (2021). Addressing the selectivity of enzyme biosensors: Solutions and perspectives. Sensors.

[49] Babadi A.A., Wan-Mohtar W.A.A.Q.I., Chang J.-S., Ilham Z., Jamaludin A.A., Zamiri G., Akbarzadeh O., Basirun W.J. (2019). High-performance enzymatic biofuel cell based on three-dimensional graphene. Int. J. Hydrogen Energy.

[50] Collier B.B., McShane M.J. (2015). Enzymatic glucose sensor compensation for variations in ambient oxygen concentration. Proc. SPIE Int. Soc. Opt. Eng..

[51] Ramanavicius A., Kausaite-Minkstimiene A., Morkvenaite-Vilkonciene I., Genys P., Mikhailova R., Semashko T., Voronovic J., Ramanaviciene A. (2015). Biofuel cell based on glucose oxidase from Penicillium funiculosum 46.1 and horseradish peroxidase. Chem. Eng. J..

[52] Zor E., Oztekin Y., Ramanaviciene A., Anusevicius Z., Bingol H., Barkauskas J., Ersoz M., Ramanavicius A. (2014). Amperometric glucose biosensor based on glucose oxidase, 1,10-phenanthroline-5,6-dione and carbon nanotubes. J. Electrochem. Soc..

[53] Krikstolaityte V., Oztekin Y., Kuliesius J., Ramanaviciene A., Yazicigil Z., Mustafa E., Okumus A., Kausaite-Minkstimiene A., Kilic Z., Solak A.O. (2013). Biofuel cell based on anode and cathode modified by glucose oxidase. Electroanalysis.

[54] Gao Z.D., Qu Y., Li T., Shrestha N.K., Song Y.-Y. (2014). Development of amperometric glucose biosensor based on prussian blue functionlized TiO_2_ nanotube arrays. Sci. Rep..

[55] Lin K.-C., Yang C.-Y., Chen S.-M., Zhao D.-H., Hou Y.-S. (2015). An interference study with different working potentials for an amperometric glucose sensor. Int. J. Electrochem. Sci..

[56] Kausaite-Minkstimiene A., Glumbokaite L., Ramanaviciene A., Dauksaite E., Ramanavicius A. (2018). An amperometric glucose biosensor based on poly(pyrrole-2-carboxylic acid)/glucose oxidase biocomposite. Electroanalysis.

[57] Jia W.-Z., Wang K., Xia X.-H. (2010). Elimination of electrochemical interferences in glucose biosensors. TrAC Trends Anal. Chem..

[58] Kulkarni T., Slaughter G. (2017). Characteristics of two self-powered glucose biosensors. IEEE Sens. J..

[59] Bhalla N., Jolly P., Formisano N., Estrela P. (2016). Introduction to biosensors. Essays Biochem..

[60] Milton R.D., Giroud F., Thumser A.E., Minteer S.D., Slade R.C.T. (2014). Bilirubin oxidase bioelectrocatalytic cathodes: The impact of hydrogen peroxide. Chem. Commun..

